# Whole Organism High-Content Screening by Label-Free, Image-Based Bayesian Classification for Parasitic Diseases

**DOI:** 10.1371/journal.pntd.0001762

**Published:** 2012-07-31

**Authors:** Ross A. Paveley, Nuha R. Mansour, Irene Hallyburton, Leo S. Bleicher, Alex E. Benn, Ivana Mikic, Alessandra Guidi, Ian H. Gilbert, Andrew L. Hopkins, Quentin D. Bickle

**Affiliations:** 1 Department of Infection and Immunity, London School of Hygiene and Tropical Medicine, London, United Kingdom; 2 Division of Biological Chemistry and Drug Discovery, College of Life Sciences, University of Dundee, Dundee, United Kingdom; 3 Accelrys Inc., San Diego, California, United States of America; 4 Accelrys Ltd, Cambridge, United Kingdom; National Institutes of Health, National Center for Advancing Translational Sciences, United States of America

## Abstract

Sole reliance on one drug, Praziquantel, for treatment and control of schistosomiasis raises concerns about development of widespread resistance, prompting renewed interest in the discovery of new anthelmintics. To discover new leads we designed an automated label-free, high content-based, high throughput screen (HTS) to assess drug-induced effects on *in vitro* cultured larvae (schistosomula) using bright-field imaging. Automatic image analysis and Bayesian prediction models define morphological damage, hit/non-hit prediction and larval phenotype characterization. Motility was also assessed from time-lapse images. In screening a 10,041 compound library the HTS correctly detected 99.8% of the hits scored visually. A proportion of these larval hits were also active in an adult worm *ex-vivo* screen and are the subject of ongoing studies. The method allows, for the first time, screening of large compound collections against schistosomes and the methods are adaptable to other whole organism and cell-based screening by morphology and motility phenotyping.

## Author Summary

Schistosomiasis is a severe helminth infection affecting an estimated 600 million people. The one drug widely available, praziquantel (PZQ), is not ideal. PZQ kills the adult worms but not the developing juveniles so the treated patient may not be cured long-term. In addition, use of repeated mass treatment campaigns with PZQ to control morbidity raises concerns about the development of drug resistance. Our work is aimed at providing starting points for drug discovery programs for schistosomiasis by screening large compound libraries against whole organisms. Praziquantel and several other known anti-schistosomal drugs are also active *in vitro* against the adult worms and the larval stages, schistosomula. The latter are ideal for novel drug screening as they can be produced in large numbers *in vitro*, are small and so are amenable to screening in microwell plates. Drug activity can be assessed visually but this is subjective and laborious. We have built an automated system for assessing drug action involving the collection of images of the larvae and the development of computer algorithms to analyze their morphology and motility, defining them as "hits" or "nonhits." The method is reliable, consistent and efficient, making it feasible, for the first time, to screen large compound collections.

## Introduction

Infection with parasitic worms (helminths) causes a huge burden of human disease [1] and economic loss to the livestock industry [2]. Currently the major control strategy for the human diseases is by large scale drug administration to schools or by mass drug administration [3]. However, the drugs available are limited in number and efficacy and their increasing use worldwide raises concerns about the development of drug resistance [4], [5].

Schistosomiasis affects an estimated 600 million people [6] but only one drug, praziquantel (PZQ), is commercially available for its treatment and control. PZQ is poorly effective against the immature worms [7] and its increasingly widespread use [8] fuels concerns about drug resistance developing [9]. There have been sporadic reports of treatment failures with PZQ [10], [11], [12] and strains isolated from such cases show lower susceptibility to PZQ [13]. However, since the development of PZQ [14] there has been limited interest in discovery of new schistosomicides apart from the recent identification of oxadiazole-2-oxides as lead compounds [15] and of anti-schistosome activities for some anti-protozoal drugs [16], [17].

The approved anthelmintics invariably were discovered by *in vivo* screening in animal models. However the low throughput and high costs of these models limits the discovery of new anti-helminth agents. Therefore new high-throughput, *in vitro* phenotypic screening methods are necessary to advance the discovery of new anthelmintics including anti-schistosome compounds. In the recent past small, focussed, compound collections have been screened against adult schistosomes recovered from rodents [18], [19]. To facilitate screening of larger compound collections microplate-based visual assays were developed using *in vitro*-derived larval stages, schistosomula, which can be generated in very large numbers [20], [21]. With a view to standardization and automation methods other than manual visual assessment have recently been applied to evaluate drug-induced damage to schistosomula [21], [22], [23], [24]. However bright-field microscopy is simpler to set up, reveals drug-specific morphological effects, and is 100% effective in detecting compounds active in the adult *ex-vivo* assays [20], [21]. To overcome the need for visual assessment we have developed a label-free, high content screen (HCS) using automatic bright-field image analysis to establish and validate a high throughput screen (HTS) for primary drug screening against schistosomes. Compound efficacy is assessed by a combination of larval motility and larval morphology quantified by Bayesian analysis. The methods make it feasible for the first time to screen very large compound collections against schistosomes and are applicable to other larval helminths.

## Materials and Methods

### Ethics statement & animals

Experimentation was carried out under the United Kingdom Animal's Scientific Procedures Act 1986 with approval from the London School of Hygiene and Tropical Medicine Ethics committee. CD1 mice supplied by Charles River, UK were maintained at St Mary's Hospital, Imperial College London.

### Parasite generation

*Schistosoma mansoni* was maintained by routine passage and schistosomula were prepared and cultured in M169 [25] as previously described [21]. Adult worm *ex-vivo* drug testing was as previously described [19].

### Compounds

The reference anti-schistosome compounds praziquantel (PZQ) and dihydroartemisinin (DHA) were obtained from Sigma-Aldrich (UK), methylclonazepam (MCZ) and Ro15-5458 (Ro15) were a gift from Dr H. Stohler (Hoffman-La Roche, Basle, Switzerland), oxamniquine (OX) was from Pfizer Ltd (Sandwich, UK) and oltipraz (OPZ) from WHO Special Programme for Research and Training in Tropical Diseases (WHO-TDR; Geneva, Switzerland). Compounds were dissolved in DMSO (Sigma-Aldrich, UK). A 10,041 compound library comprising lead-like compounds was provided by the Division of Biological Chemistry and Drug Discovery, University of Dundee.

### Plate set up

The last two columns of each test plate were reserved for controls. The test compound solvent, DMSO, was used as the negative control and added to 16 wells. Our initial testing of the image analysis models revealed that OPZ induced the lowest phenotype and motility scores reflecting the visual assessment that OLT caused the most severe effects of all of the anti-schistosome compounds tested. Therefore, OLT was chosen as the positive reference standard and applied to 8 wells. PZQ, the current therapy for schistosomiasis, induced intermediate phenotype and motility scores and so 4 wells of PZQ were included on each plate as an arbitrary check on plate performance.

Black 384-well clear-bottomed plates were selected for imaging (PerkinElmer, UK Cat no 6007460). Into each well 0.5 µl of test compound or DMSO was dry stamped using the Biomek FXp (Beckman Coulter, High Wycombe, UK). When necessary a prior intermediate dilution step in DMSO was carried out in V-bottomed dilution plates (Greiner bio-one, UK, cat no 781280). Schistosomula (120/well) were added to each well in 80 µl of M169 media using a Matrix WellMate (Thermo Scientific, Basingstoke, UK). Plates were then incubated in a Cytomat C2 automatic incubator (Thermo Scientific, UK) at 37°C, 5% CO_2_ for 3 days.

### Imaging

A Scara Robot (KiNEDx Robot KX-300-470, Peak Robotics, Colorado, USA) controlled by Overlord 3 (Process Analysis and Automation, Hemel Hempstead, UK) was used for all plate movements. After 3 days culture schistosomula were redistributed and disaggregated by using the Biomek FXp programmed to aspirate and dispense 40 µl of the well contents in each of the 4 corners of each well (×3). Bright-field images were collected using an ImageXpress^Micro^ HCS microscope (IXM; Molecular Devices, Wokingham, UK) fitted with a PhotometricsCoolSnap_HQ_ camera (Roper Scientific, Germany). Focussing of the plate and well bottom was achieved by the IXM high-speed laser auto-focus, with a 25 µm offset to focus on the larvae.

For motility analysis 5×6 sec interval time-lapse images were collected using a 4× S Fluor 0.2NA Nikon objective. For detailed morphology a 10× Ph1 Plan Fluor DL 0.3NA Nikon objective was used to collect 4 adjacent images, which were tiled together to maximise larval numbers for phenotype analysis. After imaging, the plates were visualized by two independent assessors using an inverted microscope (LeitzDiavertWetzlar, Germany).

### Statistical analysis

Differences in phenotype and motility scores were measured by one-way ANOVA with a Dunn's post-test to measure significant differences between DMSO control wells and individual drug treatments. Z factors for both the phenotype and motility scores were measured on a per plate basis in Pipeline Pilot 8.5 (Accelrys Inc., San Diego, USA) with an acceptable score being >0.5 [26].

## Results

### Development of automated image analysis

#### Production of image library

A library of ∼20,000 schistosomula images showing a wide range of drug-induced morphological effects was created by treating larvae with anti-schistosome compounds [21]: PZQ, OPZ, MCZ, Ro15, OX, DHA or culturing alone in M169 media (M169 controls) or media containing 0.625% DMSO (DMSO controls). A concentration of 10 µg/ml of the anti-schistosome compounds was chosen as this concentration induced a wide range of differing phenotypes. The images were captured from 5 plates run on different occasions with 48 wells per condition being imaged on each plate.

#### Segmentation

**Figure 1:**
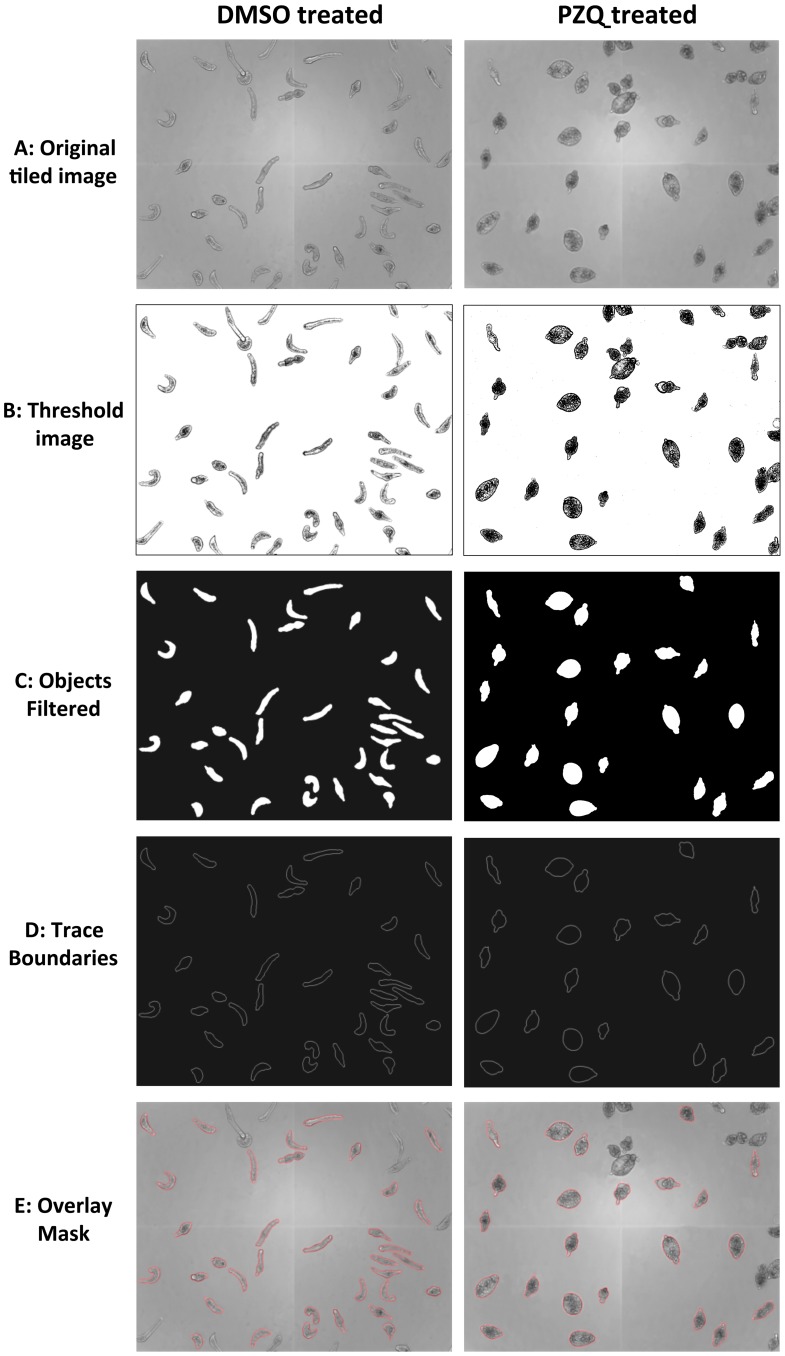
Representative example of the segmentation of control and praziquantel treated schistosomula. Images for analysis were captured on an Images Xpress^micro^ (**A**)**.** Initially an adaptive threshold was applied to each individual pixel within the image to highlight objects within the image (**B**)**.** Objects highlighted by the threshold where then closed to encapsulate whole larvae using basic greyscale morphology. For each object, background and centre points were calculated so watershed segmentation could be applied to enhance segmentation of the larvae. Filters based on area, perimeter and form factor were then applied to the mask to remove any objects too large or small to be individual larvae taking into account whether 10× or 4× images were being analysed (**C**). The individual objects were then traced (**D**) before the mask (red) was applied to the original image (**E**). The completed mask was then broken down to separate objects and applied to the original images so individual larvae could be segmented from the background.

Automated image analysis was undertaken in Pipeline Pilot 8.5 to determine differences in larval phenotype and motility after drug treatment. Initially, individual schistosomula were segmented from the background as shown in **Figure 1**. It was determined that 120 schistosomula per well gave an optimal density of larvae to allow for segmentation of sufficient non-touching parasites at 10× (morphology) and 4× (motility) magnification. The mean±SD numbers of segmented larvae per well over a 10,041 compound validation set were 29.4±8.5 at 4× and 25.3±8.8 at 10×.

#### Phenotyping protocol

The segmented larval images in the image library were manually classified as a hit (severely damaged or clearly dead) or non-hit using a custom interface (**Fig. S1**) built in Pipeline Pilot 8.5. Numerous image descriptors including pixel intensities, morphological and texture properties (**Table S1**) of the ∼20,000 segmented larvae were calculated by Pipeline Pilot 8.5. These properties were each divided into 25 bins spanning the range of values from manually phenotyped larvae. The occurrence of values in an individual bin for a given descriptor among the non-hit larvae was compared to its occurrence among the hit larvae. Descriptor, bin pairings, which were predominantly seen in just “hit” or just “non-hit” larvae, were then considered to be strong predictors of larval state. The collection of these predictors constitutes our two class Laplacian-modified Bayesian categorization model of the differences between larvae manually classified as a hit or non-hit.

A second Bayesian categorization model was similarly constructed to classify drug-specific phenotypes. In this case image descriptors of larvae treated with the specific anti-schistosome compounds were used to generate a multi-category Bayesian categorization model which predicts to which compound the larval phenotype most closely corresponds, termed ‘drug treatment class’. In any given test well the most frequent drug treatment class ascribed to segmented larvae is taken as the class for the well.

For application of the automatic image analysis to subsequent test wells, image descriptors were collected from individual segmented larvae and then processed through the Bayesian models. For each larva these models generate a larval phenotype score, and its drug treatment class. The values per well were the average phenotype score and the highest frequency treatment class respectively. Based on empirical observations data collected from a minimum of 10 segmented larvae was considered as reliable for the mean phenotype score. Wells with fewer segmented larvae are automatically rejected and reported as failures requiring manual review (see Reporting section below).

#### Validation of the phenotype model using anti-schistosome drugs

The screen was initially tested using a total of 15 plates run on several different occasions, each containing replicate columns of controls or anti-schistosome drugs. The mean±SD raw Bayesian model scores generated ranged from 1.21±1.02 for DMSO controls to −34.92±2.46 for OPZ. The phenotype score generated by this model reflects the probability that a test larva being analysed is similar to the controls entered into the model, with a score close to 0 demonstrating that the larva is very similar to the control larvae and a highly negative score demonstrating that it is very different to control larvae. It was observed that scores generated for the DMSO control wells varied a little between individual plates run at different times and with different batches of schistosomula. To compensate for such variation in the raw Bayesian model scores, individual test compound scores from a particular plate were adjusted relative to the corresponding controls on the same plate by subtracting the DMSO control mean from the test score. To avoid working with negative score values, which were incompatible with some of the software, 40 was added to all larval scores including all controls (−35 being the highest score we have ever recorded from any larva). The number derived was then divided by the mean value for the DMSO control larvae. In this way the mean value for the DMSO controls becomes 0 and the most severely disrupted, dead larvae approach −1. The adjustment of test scores relative to the DMSO controls on the same plate allowed the effects of test compounds from the same library, but tested on different plates, to be compared and ranked.

**Figure 2:**
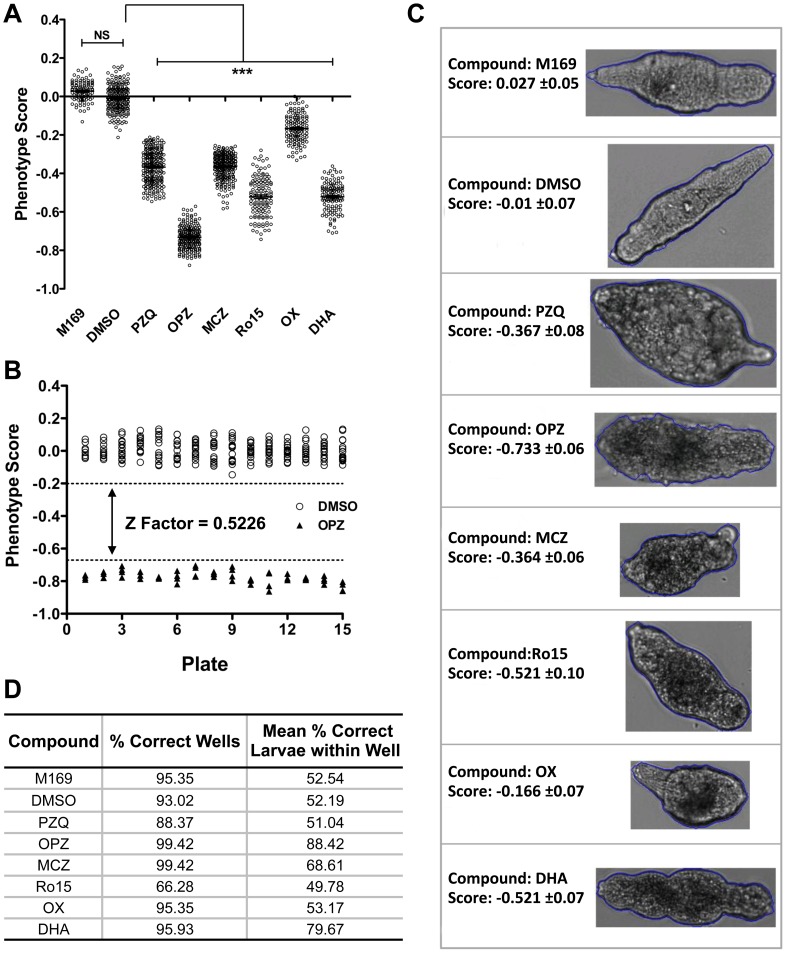
Automated image analysis of larval phenotype following treatment with anti-schistosome drugs. (**A**) Phenotype scores produced by the Bayesian prediction model from 5 replicate plates containing controls (M169/DMSO) or anti-schistosome drugs (10 µg/ml), praziquantel (PZQ), oltipraz (OPZ), methylclonazepam (MCZ), Ro15-5458 (Ro15), oxamniquine (OX) and dihydroartemisinin (DHA). N = 120 wells per condition and significance values were measured by non-parametric one-way ANOVA with individual drug treatment being compared to the DMSO controls wells by a Dunn's comparison post test, *** = p<0.001. NS; not significant. (**B**) Z factor analysis based on the distribution of the negative (DMSO; circle) and positive controls (OPZ; triangle) from 15 analyzed plates of anti-schistosome compounds (dotted lines represent the upper and lower thresholds). (**C**) Typical larval phenotypes and phenotype scores induced by the anti-schistosome drug treatments or media (M169) and solvent (DMSO) controls. (**D**) Drug treatment class prediction for wells treated with the anti-schistosome drugs and the mean number of correctly predicted larvae per well.

**Figure 2A** shows results from 5 representative plates of the 15 tested. Significant differences (p<0.001) were detected between the mean phenotype scores of the controls and each of the anti-schistosome drugs. OX had the least effect with phenotype scores overlapping with the controls. There was no such overlap for the other anti-schistosome drugs. As expected OPZ induced the lowest phenotype score (−0.73±0.06) i.e. the most severe phenotype, whilst PZQ induced a score of −0.37±0.08. Z factor analysis based on the positive (OPZ) and negative (DMSO) controls for 15 different standard test plates was evaluated to determine the robustness of the phenotype assay between different imaging days and batches of larvae. The resulting Z factor score was 0.5226 (**Fig. 2B**) demonstrating the suitability of this assay for HTS screening.

Treatment class categorization based on anti-schistosome drug larval morphology was also assessed with characteristic drug effects being shown in **Figure 2C**. With the exception of Ro15, the treatment class model successfully predicted ≥88% of wells treated with anti-schistosome compounds and on average >50% of larvae within the wells were correctly predicted (**Fig. 2D**).

#### Motility analysis protocol

**Figure 3:**
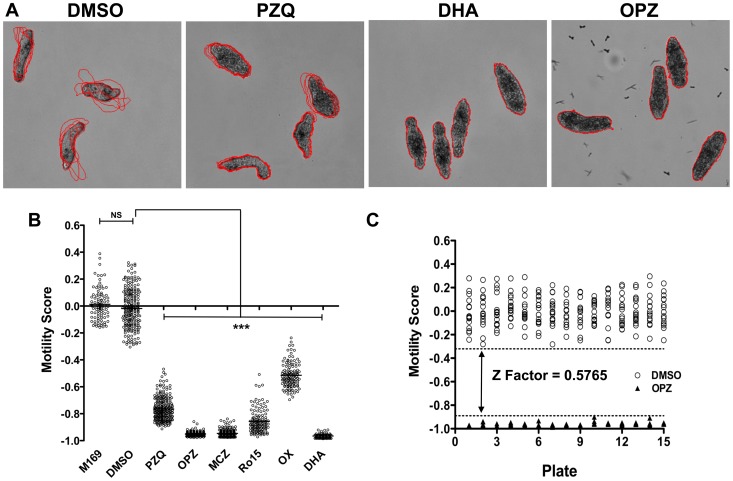
Automated image analysis of larval motility following treatment with anti-schistosome compounds. Original images were firstly segmented as described **Figure 1** and then the cumulative change in area for each individual larva measured over 5 time frames. The scores for individual larvae were averaged to generate a motility score per well. (**A**) Illustration of differences in larval motility between a high motility well (DMSO) with low overlap between larval location in successive time frames (red boundaries), a medium motility well (PZQ; praziquantel) and low motility wells with almost perfect overlap between successive time frame boundaries (DHA; dihydroartemisinin & OPZ; oltipraz). (**B**) Representative motility scores from 5 plates of anti-schistosome compounds (10 µg/ml), n = 120 wells per condition and significance values were measured by non-parametric one-way ANOVA with individual drug treatments being compared to the DMSO controls wells using a Dunn's comparison post test, *** = p<0.001. NS; not significant. (**C**) Z factor analysis based on the distribution of the negative (DMSO; open circle) and positive controls (OPZ; closed triangle) from 15 plates (dotted lines represent upper and lower thresholds).

Determination of larval motility was carried out through the analysis of time-lapse images in Pipeline pilot 8.5. Motile schistosomula frequently elongate and then round up but tend not to translocate across the well bottom so that, as also noted recently by Lee *et al.* 2012 [27], the centroids of the parasites move only small distances in a given time. Therefore, to assess motility, the cumulative change in area occupied by individual larvae between time frames was calculated (**Fig. 3A**). Larval boundaries that overlapped between successive time frames defined individual larvae. Only larvae which co-localized in 4 or 5 consecutive time frames and did not touch adjacent segmented larvae were included in the analysis. The use of 5 time-lapse images at 6 sec intervals was established empirically and reflected the balance between the need for co-localization of larvae in successive images, sufficient mean movement and overall time for imaging a well. For a given larva the cumulative change in area was divided by the average size of the larva to allow for differences in parasite size. The final motility score for a well was the average across all segmented larvae. Wells with scores from fewer than 10 schistosomula were deemed failures but were assessed manually during the reporting procedures (see below).

#### Validation of the motility model using controls and standard drugs

The motility model was assessed using the 15 plates of anti-schistosome compounds described above. The mean±SD raw motility scores ranged from 1.35±0.19 for DMSO to 0.071±0.02 for OPZ. As for the phenotype analysis, motility scores for test compounds were adjusted to compensate for plate to plate variation between the control larvae. For this, the DMSO control motility mean was subtracted from the test score and divided by the DMSO control motility mean for a given plate. **Figure 3B** shows results from 5 representative plates. Motility scores for DMSO controls were significantly (p<0.001) different from all of the anti-schistosome compounds and there was no overlap of motility scores apart from for 3 OX wells. OPZ and DHA induced the greatest effect on motility whilst PZQ treatment resulted in only a partial restriction in motility. Z factor analysis using the OPZ positive control and DMSO negative controls wells showed this assay to be suitable for high throughput screening (**Fig. 3C**) with a Z Factor score of 0.5765.

#### Combined analysis & hit threshold definition

**Figure 4:**
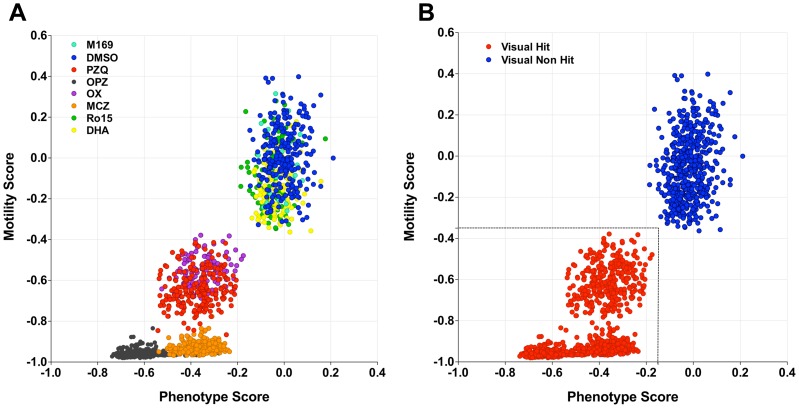
Combined phenotype and motility analysis compared with visual hit assessment for larvae treated with known anti-schistosome compounds. (**A**) Combined phenotype and motility scores for larvae treated with 10 µM praziquantel (PZQ; red), oltipraz (OPZ; black), methylclonazepam (MCZ; orange), Ro15-5458 (Ro15; blue), oxamniquine (OX; purple) dihydroartemisinin (DHA; yellow) and controls (M169; aqua & DMSO; green). (**B**) Visual assessment of hits (red) or non-hits (blue) for the wells shown in (**A**) **with a** visual hit defined as ≥70% damaged or dead schistosomula within a well [21]. Based on this, the dotted line shows the thresholds selected for definition of the hit region (−0.15 for the phenotype score and −0.35 for the motility score). N = 1500 wells across 5 plates containing anti-schistosome drugs.

Although the phenotype and motility algorithms each worked well in distinguishing drug-treated from control parasites, combined analysis of both measures has the potential to provide even more robust discrimination. **Figure 4A** shows a plot of both scores for a set of 3 plates containing anti-schistosome compounds tested at 10 µM, which is the concentration we routinely use for primary screening. The scores for all of the anti-schistosome drugs apart from DHA and Ro15 clustered distinctly from the controls with OPZ showing the most severe effects. However, the DHA and Ro15 scores were clustered with the controls, in contrast to their effects at 10 µg/ml (35.2 and 25.4 µM, respectively) which are shown in **Figures 2** **&** **3**.

All the wells treated with anti-schistosome compounds were also visually assessed for hit status based on the hit criterion of ≥70% damaged or dead larvae per well as previously described [21]. All of the wells of anti-schistosome compounds apart from DHA and Ro15 were recorded as hits (**Fig. 4B**). Based on these results, thresholds for phenotype and motility scores, which reliably delineated visual hit/non-hit wells treated with 10 µM anti-schistosome drugs, were selected as −0.15 for the phenotype and −0.35 for the motility scores (**Fig. 4B**).

We next tested the platform against a random selection of compounds which had been screened as hits/non-hits in a primary *in vitro S. mansoni* adult worm assay for the WHO-TDR Helminth Drug Initiative [19]. The larval algorithm scores for all of the adult hits fell below −0.25 phenotype and −0.8 motility scores i.e. well within the hit threshold levels (**Fig. S2**).

#### Plate reporting

An automatically-generated, interactive, reporting system was developed within Pipeline Pilot 8.5 to allow manual assessment of images and analysis scores on a per plate basis (**Fig. S3**). Reports allow the recall of images and all well scores in order to remove false positives (commonly due to crystallization of test compounds). Acceptable assay performance is assessed by confirmation of the viability of the control larvae (mean raw phenotype score >−5 and motility score >0.6) and an acceptable Z factor score of >0.5. Plates that did not conform to these scores would be considered failures.

### Validation using a 10,041 lead compound library

**Figure 5:**
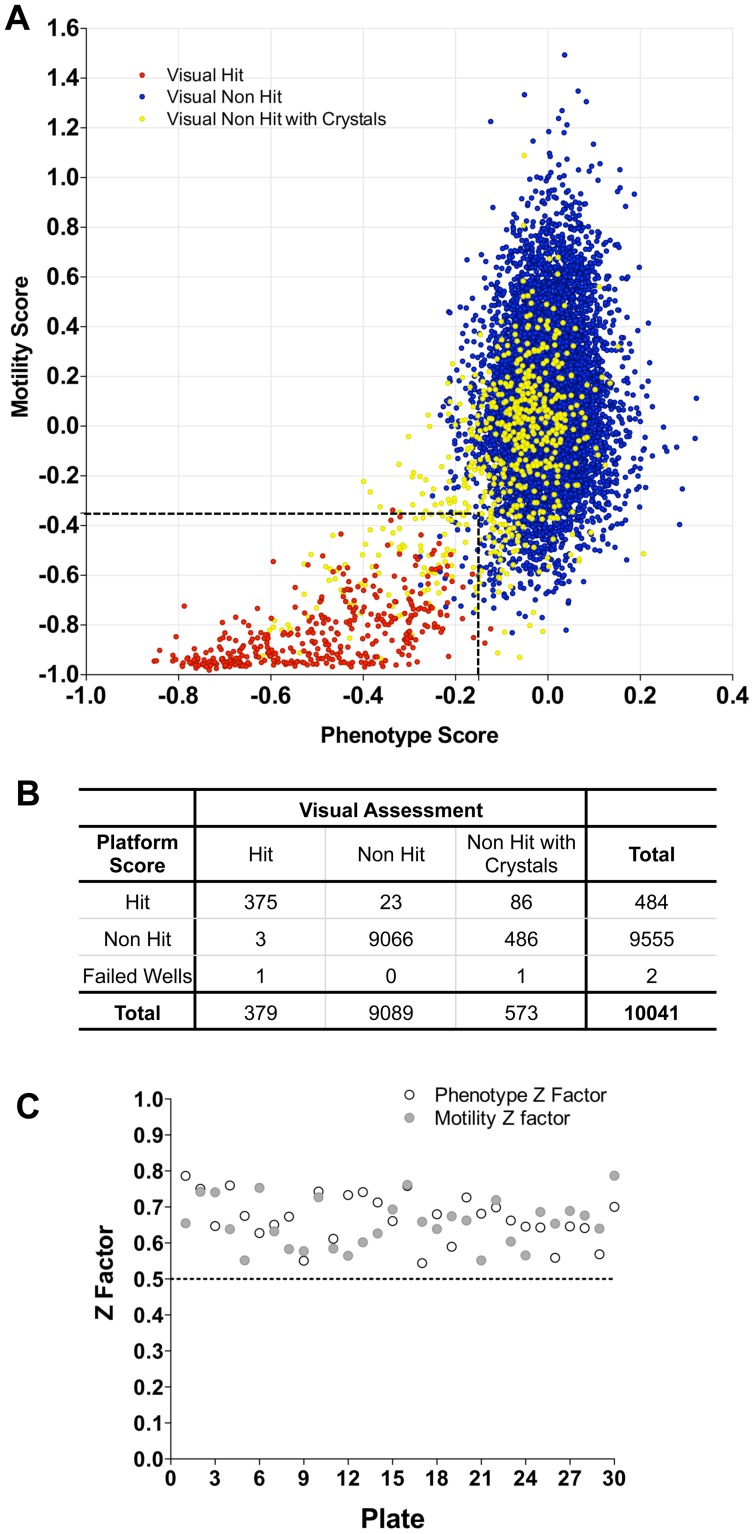
Analysis of larval phenotype and motility for the Dundee 10,041 Compound Library. (**A**) Combined phenotype and motility analysis of wells treated with 10 µM of the compounds. Visually assessed hits are shown in red, non-hits in blue and non-hits with compound crystals in the well in yellow. The hit region for automated image analysis as determined above using the anti-schistosome compounds is delineated by the dashed lines. (**B**) Performance of the Automated image analysis categorization compared to the visual assessments. (**C**) Z factor analysis of phenotype (open) and motility (closed) per plate of the Dundee compound library.

Following preliminary assessment of appropriate screening concentration/hit rate, the 10,041 compound library was screened at 10 µM. All of the plates were also visually scored by two independent assessors [21]. Using the HCS hit thresholds defined above, all the visual hits (379) apart from four were determined to be hits by the automatic analysis (**Figs. 5A & B**). Three of the failures were ascribed scores which fell just outside the hit threshold and one failed to segment due to the parasites remaining aggregated. The hit region also contained 109 wells which were visual non-hits (i.e. false positives by HCS). Of these, 86 were wells containing compound crystals. All of these were readily rejected as hits on manual review of the corresponding images in the automated HCS plate reports by marking the “Non-Hit checkbox” (**Fig. S3E**). Overall, during the manual plate reading, 780 wells were found to have crystals, of which 130 were deemed to be hits. Importantly, all of these fell in the HCS hit region. A novel phenotypic effect (internal vacuolation) was also identified during visual assessment/plate reporting for a number of compounds, a proportion of which were scored as hits by the HCS. Visually, larval viability was not considered sufficiently reduced to designate these as hits and none of the compounds were active in the adult assay. Z factor scores were reviewed to assess plate performance during screening (**Fig. 5C**) all of which were within an acceptable range.

Hits from the screen were also analysed and grouped according to larval phenotype by the Bayesian categorization model to determine which anti-schistosome compound they most resembled. From the 378 hits, 175 were ascribed to the OPZ treatment class, 60 to PZQ, 13 to MCZ, 83 to Ro15, 34 to OX and 13 to DHA.

### Repeat validation

**Figure 6:**
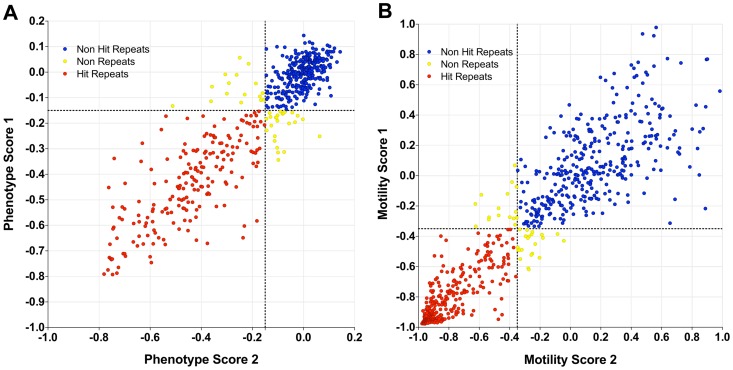
Reconfirmation screen of selected hit and non-hit compounds. Phenotype (**A**) and motility (**B**) results from initial screening (Phenotype/Motility score 1) and repeat screening (Phenotype/Motility score 2). Hits which repeated (red), non-hits which repeated (blue) and tests which did not repeat (yellow).

The assay was further validated by re-testing a selection (796) of hits and non-hits from the 10,041 compound library along with compounds from the WHO-TDR set. There was a high level of concordance between the initial and repeat testing (92.3% for morphology, **Fig. 6A**; 95.2% for motility, **Fig. 6B**).

### Secondary screening of compounds: Adult assay

**Figure 7:**
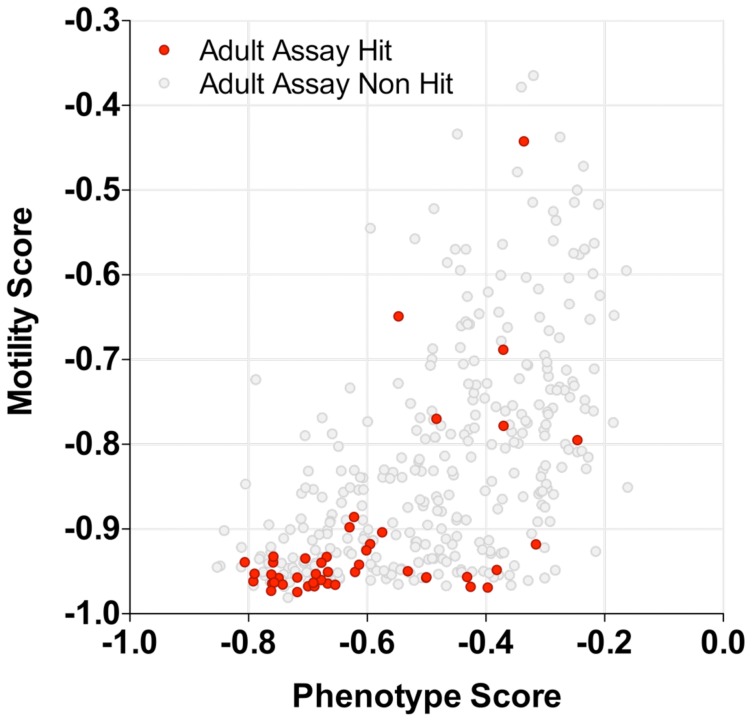
Secondary adult hit screening of larval hits for the Dundee 10,041 Compound Library. Larval phenotype and motility scores for the compounds which were tested in the secondary adult assay. Adult hits are depicted in red and non-hits in grey.

*In vitro* testing against *ex-vivo* adult worms is a crucial secondary screen since the adult worm is the key target of drug action. Preliminary testing of a few of the larval hits in the secondary adult worm assay [19] at 10 µM yielded a very low hit rate and so the hits were all tested at 20 µM which gave 45 adult hits. Plotting the larval phenotype and motility scores for these hits (**Fig. 7**) showed that the majority corresponded to severe larval phenotypes but a few were scattered throughout the hit threshold region. The number of hits ascribed to different treatment classes were OPZ 28, PZQ 6, DHA 4, Ro15 4, OX 2 and MCZ 1. Subsequent IC_50_ testing of the adult hits identified 7 compounds which had IC50s of <10 µM and which are the subject of on-going studies. These compounds were attributed the drug treatment classes OLT 5, PZQ 1, OX 1.

## Discussion

Whole organism screens have an advantage over more target-based approaches as hit compounds can be directly translated into new therapeutics and it has been suggested that the lack of these screens has impacted on the discovery of new compounds [28]. We have established the first HTS for whole organism screening of helminths based on bright-field HCS analysis of morphological and motility changes in schistosome larvae. The published examples of primary high content drug screens are predominately cell-based [29], [30]. A few involving whole organism screens for *Caenorhabditis elegans* [31], zebrafish embryos [32], *Leishmania major* [33] and *Plasmodium falciparum* [34] have been reported. These exploit use of fluorescent probes or proteins since fluorescence provides more contrast, sharpness and discrimination compared with transmitted-light imaging. However, use of fluorescent probes often involves more manipulations and use of potentially toxic fluorophores [35]. Furthermore, fluorescent transgenic lines are not available for certain organisms of interest including parasitic helminths.

Transmitted-light imaging and analysis has been developed for whole well segmentation of *C. elegans* [36] and to demonstrate drug-induced motility changes using optical flow in adult *Brugia malayi* [37]. Our approach focussed on the development of bright-field imaging of morphology and motility of larval schistosomes directly comparable to the manual visualization used previously [20], [21]. This requires segmentation and analysis of whole organisms as has been described for *C. elegans* [31], [38], [39]. Our segmentation method differs from previously reported approaches applied to schistosomula, which used a single threshold and aimed to segment touching larvae [40], [41]. The protocol we describe uses an adaptive threshold (relative to regional background intensity) and avoids the need to segment touching organisms due to successful larval resuspension prior to imaging. This resulted in capture of sufficient individual larvae (≥10/well) at 4× and 10× for analysis in 97.8% wells from the 10,041 compound library.

Analysis of cells or whole organisms by HCS has the potential to generate image profiles to define characteristic drug phenotypes [42] which may ultimately be interpretable in relation to compound activity, modes of action and molecular targets, currently undefined for any of the known schistosomicides [43]. Different schistosomicides induce a range of distinct morphological effects in both adults [19] and larvae [20] which led us to develop the treatment class model, grouping test compounds causing similar effects to the anti-schistosome drugs. In screening the 10,041 compound library, larval treatment classes ascribed to adult hits with IC_50_<10 µM were OLT 5, PZQ 1, OX 1. Using an alternative approach to our Bayesian treatment class model, agglomerative hierarchical clustering or DBSCAN has been recently developed [41] to ascribe larval phenotypes according to defined morphological classes. The utility of such classifications models according to phenotype may become clear as more hits are detected and structure/activity relationships are understood.

The Bayesian models developed here can also be readily modified by uploading larval images of any novel phenotypic effects identified during image review for any compounds of particular interest e.g. those which show adult worm activity. Similarly the models could be modified by addition of images of novel phenotypes deemed on manual review not to warrant hit status e.g. those causing internal vacuolation identified during our screening.

The HCS was validated using several compound collections. At 10 µM, a commonly used concentration for primary screening, the models reliably and reproducibly distinguished all of our reference schistosomicides from the controls with the exception of DHA and Ro15. This is not considered a failure of the primary assay since both of these compounds are inactive in the visual larval and adult worm *in vitro* assays at 10 µM. Based on the results with the anti-schistosome drugs, hit thresholds of −0.15 for phenotype and −0.35 for motility were established and proved robust in detecting 40 previously tested adult hit compounds [19] and 99.8% of visually assessed hits from the 10,041 compound library. The HCS produced a false positive rate of 1.1%, mostly (78%) due to the precipitation of test compounds in wells which in fact contained healthy parasites and which were readily redefined as non-hits during routine reviewing of the automated plate reports.

The HCS offers significant advantages over other recent approaches to objective quantitation of schistosomula damage. Peak *et al.* (2010) [23] successfully assessed severe drug effects based on uptake of fluorescent markers but the assay involves multiple wash steps and was unable to detect more subtle effects e.g. caused by PZQ. An assay based on use of the redox indicator, Alamar Blue, was similarly less sensitive than visual assessment for more subtle effects and was influenced by variation in parasite numbers per well [21]. Isothermal microcalorimetry [44], assessment of motility via electrical impedance measurement [45] and optical flow [37] are also able to quantitate drug effects in schistosomes, but are not currently readily adaptable to high throughput applications.

Much of the HTS described is automated and simple to operate. Once test plates have been set up and left for 3 days in the Cytomat incubator, the image capture and analysis systems would run automatically after opening “Overlord 3”, the automation control software and pressing “run”. Thereafter, plates emerge from the Cytomat and are robotically moved around for barcode reading, parasite resuspension, imaging and then return to the cytomat. Subsequent analysis involves opening Pipeline Pilot 8.5 and running the analysis protocol. Once complete the plate reports are accessible within an intranet web-port. To become sufficiently familiar with the current customized system would require only a couple of days of training and completing a few test runs. Basic modification of the analysis protocols e.g. to run fewer test compounds in a plate, would require some familiarity with Pipeline Pilot 8.5 as well as MetaXpress software which controls the IXM microscope. More significant alterations of the protocols would require in-depth knowledge of all the different software involved. Currently the platform takes 2 hrs to image a 384 well plate and a further 2 hrs to analyse the phenotype and motility images. The system can be programmed to start imaging at any time of the day and could run close to continuously. So if test plates were set up on each of 4 days per week, the throughput, limited by plate reading, would be ∼48 plates or 16,896 compounds/week which would require around 2×10^6^ cercariae/week. In fact it is cercarial production which is limiting our current throughput capability to around 10 plates twice per week (∼7,000 compounds/week, 350,000/year).

In conclusion, the HCS described is suitable for primary screening of large compound collections for activity against schistosomes. Further studies are ongoing to adapt this system to screen against several species of nematode larvae of medical and veterinary importance, which may allow parallel testing of libraries against various helminths.

## Supporting Information

Figure S1 **Schistosoma Analysis Platform custom interface.** The custom interface enables selection and viewing of a particular plate (**A**) and well (**B**). This subsequently loads individual wells (**C**) and segmented larval images (**D**). Each segmented larva can be selected (**E**) and manually phenotyped with a selection of criteria (**F**) along with the anti-schistosome compound with which the well was treated.
(TIF)

Figure S2 **Combined larval phenotype and motility analysis of compounds initially screened against adult worms.** Larval phenotype and motility scores for compounds from a previously tested WHO/TDR compound library which were adult hits (red) or non hits (blue) (N = 40 of each).
(TIF)

Figure S3 **Interactive plate report.** Data from a chosen plate is displayed in the interactive web port via a tabbed table, bar charts and a phenotype/motility scatter plot (**A**)**.** Data from a particular well can be highlighted in the table or on the scatter plot by clicking on a cell in the table or a point on the graph. This will also display the appropriate phenotype and motility images in the dynamic container (**B**)**.** Clicking on these enlarges them and also shows images and phenotype scores of the individual larvae (**C**) or motility scores for individual larvae (**D**)**.** Once a well has been assessed to be a hit or non-hit, the table can be updated by clicking the appropriate check box (**E**) which once submitted (**F**) will update the database.
(TIF)

Table S1 **Image descriptors used to build Bayesian models.** Image descriptors including morphological properties (size, solidity, circularity, eccentricity) pixel intensity properties (normalized intensity mean, standard deviation, skewness, etc.) and texture properties at multiple length scales (spatial moments, central moments, Hu moments, co-occurrence correlation, co-occurrence entropy etc.) were used to build both Bayesian models for phenotype scoring and phenotype classification.
(TIF)

## References

[1] Hotez PJ, Brindley PJ, Bethony JM, King CH, Pearce EJ, et al (2008) Helminth infections: the great neglected tropical diseases. The Journal of clinical investigation 118: 1311–1321. 18382743 10.1172/JCI34261 PMC2276811

[2] Knox MR, Besier RB, Le Jambre LF, Kaplan RM, Torres-Acosta JF, et al (2011) Novel approaches for the control of helminth parasites of livestock VI: Summary of discussions and conclusions. Veterinary parasitology 10.1016/j.vetpar.2011.11.054 22154257

[3] WHO (2010) Working to overcome the global impact of neglected tropical diseases. WHO/HTM/NTD/20101 Geneva, 2010.

[4] WHO (2011) Working to overcome the global impact of neglected tropical diseases. Update 2011. WHO/HTM/NTD/20113 Geneva, 2011.

[5] Geary TG, Woo K, McCarthy JS, Mackenzie CD, Horton J, et al (2010) Unresolved issues in anthelmintic pharmacology for helminthiases of humans. International Journal for Parasitology 40: 1–13. 19932111 10.1016/j.ijpara.2009.11.001

[6] King CH (2010) Parasites and poverty: the case of schistosomiasis. Acta Trop 113: 95–104. 19962954 10.1016/j.actatropica.2009.11.012 PMC2812649

[7] Sabah AA, Fletcher C, Webbe G, Doenhoff MJ (1986) Schistosoma mansoni: chemotherapy of infections of different ages. Experimental parasitology 61: 294–303. 3086114 10.1016/0014-4894(86)90184-0

[8] Fenwick A, Webster JP, Bosque-Oliva E, Blair L, Fleming FM, et al (2009) The Schistosomiasis Control Initiative (SCI): rationale, development and implementation from 2002–2008. Parasitology 136: 1719–1730. 19631008 10.1017/S0031182009990400

[9] Cioli D (2000) Praziquantel: is there real resistance and are there alternatives? Curr Opin Infect Dis 13: 659–663. 11964838 10.1097/00001432-200012000-00014

[10] Stelma FF, Talla I, Sow S, Kongs A, Niang M, et al (1995) Efficacy and side effects of praziquantel in an epidemic focus of Schistosoma mansoni. American Journal of Tropical Medicine and Hygiene 53: 167–170. 7677219 10.4269/ajtmh.1995.53.167

[11] Guisse F, Polman K, Stelma FF, Mbaye A, Talla I, et al (1997) Therapeutic evaluation of two different dose regimens of praziquantel in a recent Schistosoma mansoni focus in Northern Senegal. American Journal of Tropical Medicine and Hygiene 56: 511–514. 9180600 10.4269/ajtmh.1997.56.511

[12] Ismail M, Metwally A, Farghaly A, Bruce J, Tao LF, et al (1996) Characterization of isolates of Schistosoma mansoni from Egyptian villagers that tolerate high doses of praziquantel. American Journal of Tropical Medicine and Hygiene 55: 214–218. 8780463 10.4269/ajtmh.1996.55.214

[13] Cioli D, Botros SS, Wheatcroft-Francklow K, Mbaye A, Southgate V, et al (2004) Determination of ED50 values for praziquantel in praziquantel-resistant and -susceptible Schistosoma mansoni isolates. International Journal for Parasitology 34: 979–987. 15217737 10.1016/j.ijpara.2004.05.001

[14] Caffrey CR, Secor WE (2011) Schistosomiasis: from drug deployment to drug development. Curr Opin Infect Dis 10.1097/QCO.0b013e328349156f 21734570

[15] Sayed AA, Simeonov A, Thomas CJ, Inglese J, Austin CP, et al (2008) Identification of oxadiazoles as new drug leads for the control of schistosomiasis. Nat Med 14: 407–412. 18345010 10.1038/nm1737 PMC2700043

[16] Eissa MM, El-Azzouni MZ, Amer EI, Baddour NM (2011) Miltefosine, a promising novel agent for schistosomiasis mansoni. International Journal for Parasitology 41: 235–242. 21055404 10.1016/j.ijpara.2010.09.010

[17] Keiser J, Manneck T, Vargas M (2011) Interactions of mefloquine with praziquantel in the Schistosoma mansoni mouse model and in vitro. The Journal of antimicrobial chemotherapy 66: 1791–1797. 21602552 10.1093/jac/dkr178

[18] Hudson A, Nwaka S (2007) The Concept Paper on the Helminth Drug Initiative. Onchocerciasis/lymphatic filariais and schistosomiasis: opportunities and challenges for the discovery of new drugs/diagnostics. Expert Opinion on Drug Discovery 2: S3–S7. 23489030 10.1517/17460441.2.S1.S3

[19] Ramirez B, Bickle Q, Yousif F, Fakorede FK, Mouries M-A, et al (2007) Schistosomes: challenges in compound screening. Expert Opinion on Drug Discovery 2: S53–361. 23489033 10.1517/17460441.2.S1.S53

[20] Abdulla MH, Ruelas DS, Wolff B, Snedecor J, Lim KC, et al (2009) Drug discovery for schistosomiasis: hit and lead compounds identified in a library of known drugs by medium-throughput phenotypic screening. PLoS neglected tropical diseases 3: e478. 19597541 10.1371/journal.pntd.0000478 PMC2702839

[21] Mansour NR, Bickle QD (2010) Comparison of microscopy and Alamar blue reduction in a larval based assay for schistosome drug screening. PLoS neglected tropical diseases 4: e795. 20706580 10.1371/journal.pntd.0000795 PMC2919390

[22] Holtfreter MC, Loebermann M, Frei E, Riebold D, Wolff D, et al (2010) Schistosomula, pre-adults and adults of Schistosoma mansoni ingest fluorescence-labelled albumin in vitro and in vivo: implication for a drug-targeting model. Parasitology 137: 1645–1652. 20500919 10.1017/S0031182010000405

[23] Peak E, Chalmers IW, Hoffmann KF (2010) Development and validation of a quantitative, high-throughput, fluorescent-based bioassay to detect schistosoma viability. PLoS neglected tropical diseases 4: e759. 20668553 10.1371/journal.pntd.0000759 PMC2910722

[24] Manneck T, Braissant O, Ellis W, Keiser J (2011) Schistosoma mansoni: antischistosomal activity of the four optical isomers and the two racemates of mefloquine on schistosomula and adult worms in vitro and in vivo. Experimental parasitology 127: 260–269. 20732321 10.1016/j.exppara.2010.08.011

[25] Basch PF (1981) Cultivation of Schistosoma mansoni in vitro. I. Establishment of cultures from cercariae and development until pairing. The Journal of parasitology 67: 179–185. 7241277

[26] Zhang JH, Chung TD, Oldenburg KR (1999) A Simple Statistical Parameter for Use in Evaluation and Validation of High Throughput Screening Assays. J Biomol Screen 4: 67–73. 10838414 10.1177/108705719900400206

[27] Lee H, Moody-Davis A, Saha U, Suzuki BM, Asarnow D, et al (2012) Quantification and clustering of phenotypic screening data using time-series analysis for chemotherapy of schistosomiasis. BMC genomics 13 Suppl 1: S4. 10.1186/1471-2164-13-S1-S4 PMC3471343 22369037

[28] Swinney DC, Anthony J (2011) How were new medicines discovered? Nature reviews Drug discovery 10: 507–519. 21701501 10.1038/nrd3480

[29] Bickle M (2010) The beautiful cell: high-content screening in drug discovery. Analytical and bioanalytical chemistry 398: 219–226. 20577725 10.1007/s00216-010-3788-3

[30] Zanella F, Lorens JB, Link W (2010) High content screening: seeing is believing. Trends in biotechnology 28: 237–245. 20346526 10.1016/j.tibtech.2010.02.005

[31] Gosai SJ, Kwak JH, Luke CJ, Long OS, King DE, et al (2010) Automated high-content live animal drug screening using C. elegans expressing the aggregation prone serpin alpha1-antitrypsin Z. PloS one 5: e15460. 21103396 10.1371/journal.pone.0015460 PMC2980495

[32] Peravali R, Gehrig J, Giselbrecht S, Lutjohann DS, Hadzhiev Y, et al (2011) Automated feature detection and imaging for high-resolution screening of zebrafish embryos. Biotechniques 50: 319–324. 21548893 10.2144/000113669

[33] Siqueira-Neto JL, Song OR, Oh H, Sohn JH, Yang G, et al (2010) Antileishmanial high-throughput drug screening reveals drug candidates with new scaffolds. PLoS neglected tropical diseases 4: e675. 20454559 10.1371/journal.pntd.0000675 PMC2864270

[34] Baniecki ML, Wirth DF, Clardy J (2007) High-throughput Plasmodium falciparum growth assay for malaria drug discovery. Antimicrobial agents and chemotherapy 51: 716–723. 17116676 10.1128/AAC.01144-06 PMC1797774

[35] Graves R (2011) Incorporating transmitted light modalities into high-content analysis assays. In: Label-free technologies for drug discovery. CMaM LM, editor. New York: John Wiley and Sons, Ltd. 101 = 110.

[36] Moy TI, Conery AL, Larkins-Ford J, Wu G, Mazitschek R, et al (2009) High-throughput screen for novel antimicrobials using a whole animal infection model. ACS Chem Biol 4: 527–533. 19572548 10.1021/cb900084v PMC2745594

[37] Marcellino C, Gut J, Lim KC, Singh R, McKerrow J, et al (2012) WormAssay: a novel computer application for whole-plate motion-based screening of macroscopic parasites. PLoS neglected tropical diseases 6: e1494. 22303493 10.1371/journal.pntd.0001494 PMC3269415

[38] Stephens GJ, Johnson-Kerner B, Bialek W, Ryu WS (2008) Dimensionality and dynamics in the behavior of C. elegans. PLoS computational biology 4: e1000028. 18389066 10.1371/journal.pcbi.1000028 PMC2276863

[39] Cronin CJ, Mendel JE, Mukhtar S, Kim YM, Stirbl RC, et al (2005) An automated system for measuring parameters of nematode sinusoidal movement. BMC genetics 6: 5. 15698479 10.1186/1471-2156-6-5 PMC549551

[40] Singh R, Pittas MI, Heskia I, Xu F, McKerrow JH, et al (2009) Automated image-based phenotypic screening for high-throughput drug discovery. In: Proceedings of 22nd IEEE Symposium on Computer-Based Medical Systems, 2009: ALbuquerque, New Mexico, USA.

[41] Lee H, Moody-Davis A, Saha U, Suzuki B, Asarnow D, et al (2012) Quantification and clustering of phenotypic screening data using time-series analysis for chemotherapy of schistosomiasis. BMC Genomics 13: S4. 10.1186/1471-2164-13-S1-S4 PMC3471343 22369037

[42] Perlman ZE, Slack MD, Feng Y, Mitchison TJ, Wu LF, et al (2004) Multidimensional drug profiling by automated microscopy. Science 306: 1194–1198. 15539606 10.1126/science.1100709

[43] Doenhoff MJ, Cioli D, Utzinger J (2008) Praziquantel: mechanisms of action, resistance and new derivatives for schistosomiasis. Curr Opin Infect Dis 21: 659–667. 18978535 10.1097/QCO.0b013e328318978f

[44] Manneck T, Braissant O, Haggenmuller Y, Keiser J (2011) Isothermal microcalorimetry to study drugs against Schistosoma mansoni. Journal of clinical microbiology 49: 1217–1225. 21270220 10.1128/JCM.02382-10 PMC3122815

[45] Smout MJ, Kotze AC, McCarthy JS, Loukas A (2010) A novel high throughput assay for anthelmintic drug screening and resistance diagnosis by real-time monitoring of parasite motility. PLoS neglected tropical diseases 4: e885. 21103363 10.1371/journal.pntd.0000885 PMC2982823

