# Supplementary figures and images for: Whole Organism High-Content Screening by Label-Free, Image-Based Bayesian Classification for Parasitic Diseases
Source: PLoS Negl Trop Dis. 2012 Jul 31;6(7):e1762. doi: 10.1371/journal.pntd.0001762 (PMC3409125; doi:10.1371/journal.pntd.0001762)

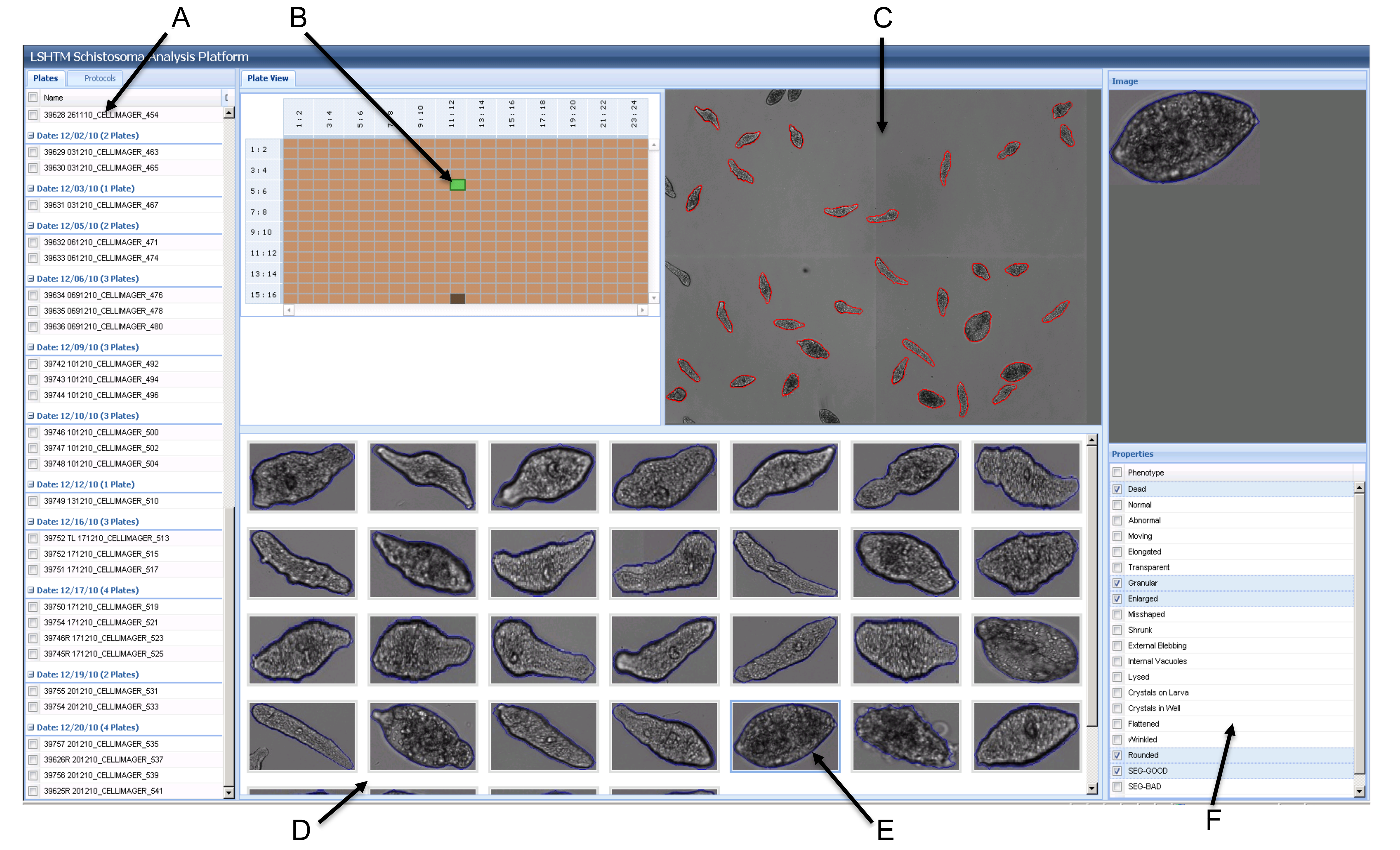

Supplement: Figure S1 — Schistosoma Analysis Platform custom interface. The custom interface enables selection and viewing of a particular plate (A) and well (B). This subsequently loads individual wells (C) and segmented larval images (D). Each segmented larva can be selected (E) and manually phenotyped with a selection of criteria (F) along with the anti-schistosome compound with which the well was treated. (TIF) [file pntd.0001762.s001.tif]

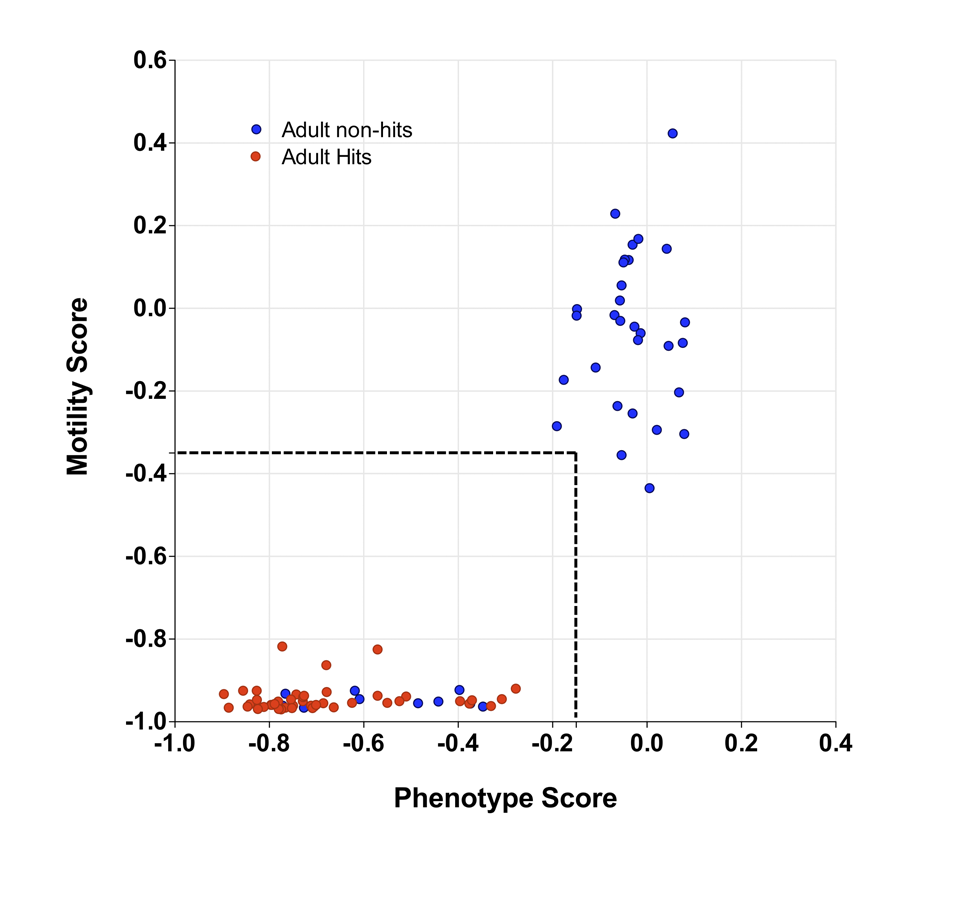

Supplement: Figure S2 — Combined larval phenotype and motility analysis of compounds initially screened against adult worms. Larval phenotype and motility scores for compounds from a previously tested WHO/TDR compound library which were adult hits (red) or non hits (blue) (N = 40 of each). (TIF) [file pntd.0001762.s002.tif]

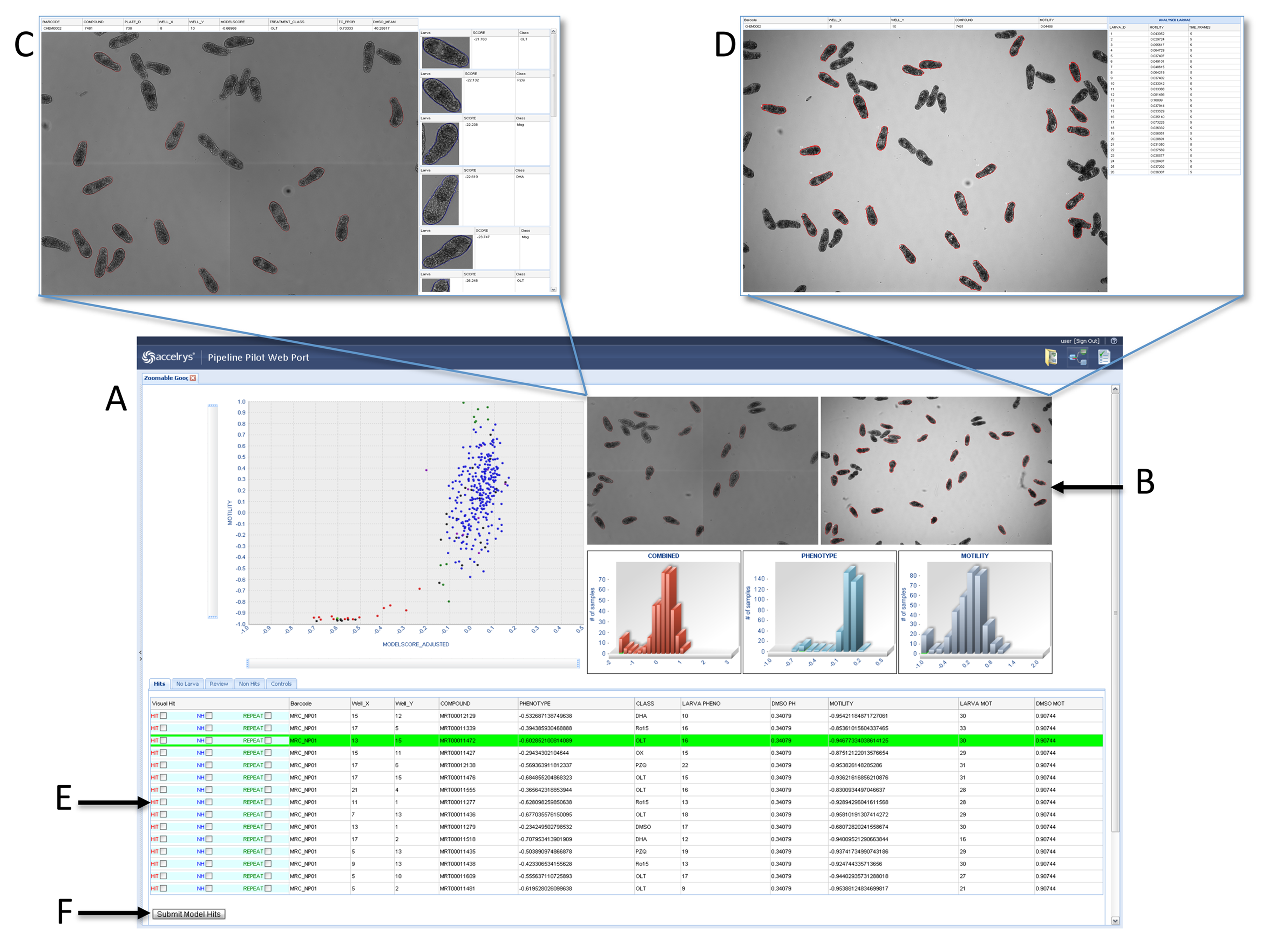

Supplement: Figure S3 — Interactive plate report. Data from a chosen plate is displayed in the interactive web port via a tabbed table, bar charts and a phenotype/motility scatter plot (A). Data from a particular well can be highlighted in the table or on the scatter plot by clicking on a cell in the table or a point on the graph. This will also display the appropriate phenotype and motility images in the dynamic container (B). Clicking on these enlarges them and also shows images and phenotype scores of the individual larvae (C) or motility scores for individual larvae (D). Once a well has been assessed to be a hit or non-hit, the table can be updated by clicking the appropriate check box (E) which once submitted (F) will update the database. (TIF) [file pntd.0001762.s003.tif]

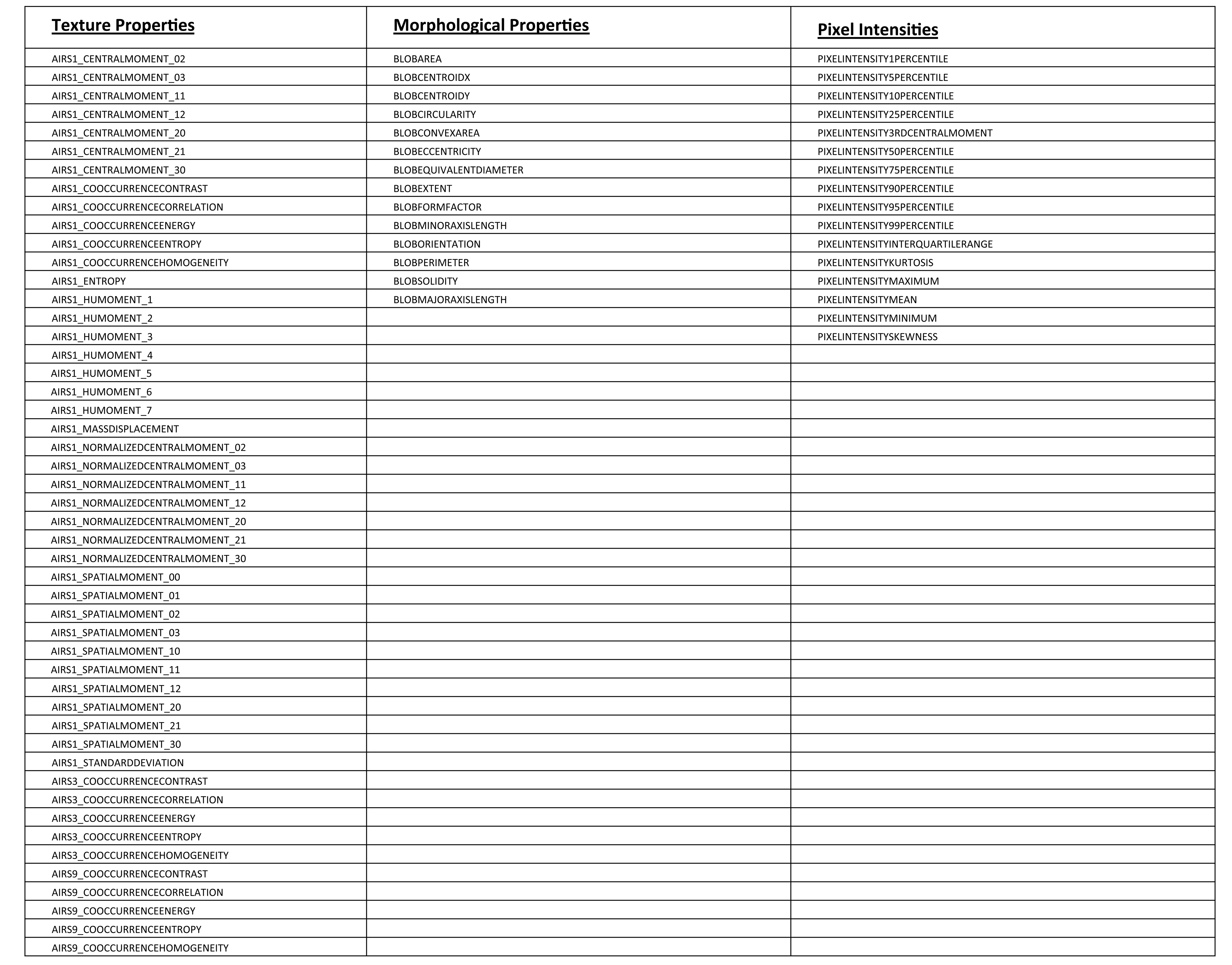

Supplement: Table S1 — Image descriptors used to build Bayesian models. Image descriptors including morphological properties (size, solidity, circularity, eccentricity) pixel intensity properties (normalized intensity mean, standard deviation, skewness, etc.) and texture properties at multiple length scales (spatial moments, central moments, Hu moments, co-occurrence correlation, co-occurrence entropy etc.) were used to build both Bayesian models for phenotype scoring and phenotype classification. (TIF) [file pntd.0001762.s004.tif]
